# Benign Paroxysmal Torticollis

**DOI:** 10.3390/life14060717

**Published:** 2024-05-31

**Authors:** Elisabetta Tozzi, Luca Olivieri, Pamela Silva

**Affiliations:** Department of Clinical Medicine, Public Health, Life and Environmental Sciences, University of Studies of L’Aquila, 67010 L’Aquila, Italy; luca.olivieri@student.univaq.it (L.O.); pamela.silva@student.univaq.it (P.S.)

**Keywords:** torticollis, children periodic syndrome, migraine genetics

## Abstract

Background: The purpose of this review is to clarify the natural course of benign paroxysmal torticollis (BPT) and update the information on the relationship of this disorder with migraine. BPT belongs to a group of “episodic syndromes that may be associated with migraine” and is diagnosed according to diagnostic criteria of the International Classification of Headache Disorders, 3rd edition. BPT affects infants and young children and is often an underdiagnosed manifestation since it is not recognized in cases with a benign evolution, requiring a careful differential diagnosis. It was first described by Snyder in 1969 as a movement disorder, a cervical dystonia consequent to labyrinthic disorder. Materials and methods: The PubMed and Web of Science databases were consulted from 1968 to 2024, according to the Preferred Reporting Items for Systematic Reviews and Meta-Analyses (PRISMA) 2020 guidelines. Results: In total, 113 articles were identified, 86 selected, and 25 considered for the purpose of this review. Clinical studies were considered in relation to evolution, cognitive, and motor development; genetic and not genetic etiology; the relationship with migraine with and without aura; vestibular migraine; hemiplegic migraine; and paroxysmal vertigo.

## 1. Introduction

Benign paroxysmal torticollis (BPT) is a movement disorder that can be classified according to neurological semeiotics such as cervical dystonia [1]. BPT in infancy was first described by Snyder in 1969 [2] as recurrent episodes of an abnormal rotation and inclination of the head to one side and was classified as a vestibular disorder. The reason for this was that the attacks of BPT observed in four patients developed into benign paroxysmal vertigo (BPVC) in subsequent years [1,2]. Afterwards, some authors postulated that BPT could be due to vestibular disorders in the central vestibular region or vestibule–cerebellar connections, especially when associated with ataxia [3]. However, to date, the etiology of BPT is not certain. There are relatively few reports on this functional disorder. In fact, only about 150 cases have been reported in the literature since 1969 [2]. Moreover, it was described as a rare disease on the ORPHANET website [4]. The presence of a physician is not always required for BPT treatment since BPT is often a benign event with a favorable prognosis. For this reason, the doctor may not always check for the recurrence of paroxysmal episodes, which are very important for the diagnosis of BPT [5,6,7]. BPT is considered a rare disease and is described on the ORPHANET website [4], where the prevalence of the disease is reported to be <1/1,000,000. In the previous International Classification of Headache Disorders (ICHD-II) of 2004 [8], BPT was reported in the chapter “Periodic childhood syndromes that are commonly pre-cursors of migraine”. It was subsequently moved from the “Appendix” section to the body of the beta version of ICHD-III [9]. Now, in the current classification ICHD-III, 2018 [10], “Periodic Child Syndromes”, it is referred to as “Episodic Syndromes that may be associated with migraine”. Chapter 1.6 mentions cyclic vomiting syndrome (CVS), abdominal migraine (AM), benign paroxysmal vertigo in childhood (BPVC), and, in point 1.6.3, BPT. Childhood episodic syndromes are characterized by reversible and stereotypical attacks with periodic recurrence. Children are healthy and neurologically normal in the attack-free interval. 

The purpose of this review was to evaluate the manifestation of the “migraine” condition of BPT through a comparison of case studies. Moreover, we wanted to evaluate the natural history of the disorder through a comparison and evaluation of longitudinal studies.

## 2. Materials and Methods

First, we started by searching the PubMed and Web of Science databases for all articles published between 1968 and 2024. Second, the following filters were placed: etiology and genetics and childhood periodic syndrome. The search and choice of studies were made according to the Preferred Reporting Items for Systematic Reviews and Meta-Analyses (PRISMA) 2020 guidelines [11]. We analyzed all the studies focusing on topics considering the following features: predisposing factors, familiarity, etiology, genetics, clinical features, possible pathogenetic mechanisms, and treatment. Benign paroxysmal torticollis (BPT) was linked to migraine and childhood periodic syndrome. The selection process included the following types of articles: case reports, longitudinal and observational studies, and comparative studies. Reviews, systematic reviews, reviews of case reports, and articles not in English were excluded.

## 3. Results

We examined a total of 114 articles, 53 recruited by PubMed and 61 by Web of Science. Among the 114 articles, 25 were reviews. A total of 28 articles were excluded because they were duplicates in the two databases and 8 articles were excluded because they were off-topic. Case reports, epidemiological and genetics studies, and longitudinal studies were included. The articles taken into consideration for the purpose of this review were the following: six longitudinal studies [1,12,13,14,15,16], seven genetic studies [14,17,18,19,20,21,22], and eight case reports [2,5,6,7,23,24,25,26].

**Figure 1:**
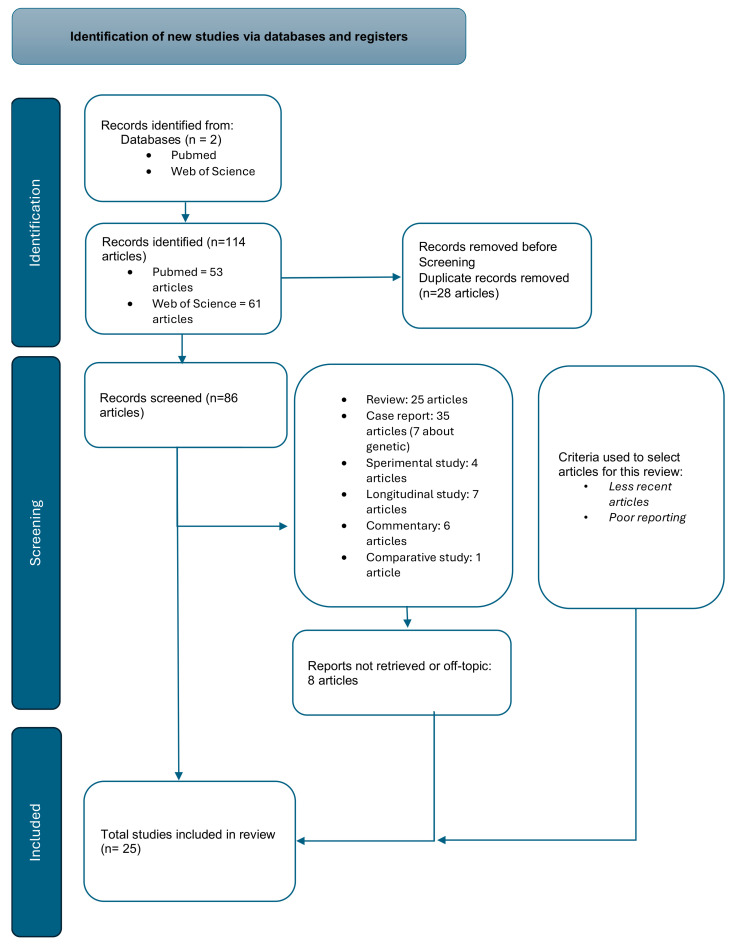
PRISMA flow chart.

Figure 1 shows the Prisma flow chart.

These studies were published over a long period of time, from 1968 to 2022. This was due to the rarity of benign paroxysmal torticollis (BPT). Most studies referred to reports with very few cases, except for the studies by Drigo [6], Moavero [26], and Greene [5] which reported a series of more than 20 subjects followed over time. Our review evaluated 273 cases described by 15 authors, a higher number than in previous reviews on this topic. On the basis of these reports, it was possible to outline the clinical manifestations of BPT and its evolution.

- Epidemiology

The prevalence of BPT is difficult to establish. As already mentioned in the introduction, the diagnosis of BPT is underestimated and, therefore, its real prevalence and epidemiology are difficult to evaluate. In this regard, the study by Al-Twaijri [12] is very interesting. The author, out of a total of 5848 patients from the general pediatric neurology outpatient database, found 1106 patients with migraine and 108 patients with equivalent migraines (1.8% of the total, 9.8% of migraine sufferers). The author considered as migraine equivalents the following disorders: benign paroxysmal vertigo of childhood (BPVC), acephalgic migraine, BPT, abdominal migraine/cyclical vomiting, and acute confusional migraine. Among migraine equivalents, BPT was present in 11 children (10.2% of patients with migraine equivalents). Another retrospective study showed that up to 70% of children with primary headaches had a previous history of one or more of the episodic syndromes [22].

- Clinical data

Snyder [2] described 12 cases of BPT in infants beginning at 2–8 months of age. The attacks were variably recurrent and self-limiting, without premonitory symptoms. The infants developed a head tilt with head rotation. Some had no other symptoms; others showed paleness, agitation, irritability, and vomiting. Episodes could last hours or days. There did not appear to be any repeats. The attacks were spontaneous and stereotyped. They occurred 2–3 times a month. No predisposing events were reported. There was no loss of consciousness and/or awareness and the attacks could remain unilateral or alternating. Torticollis could be associated with tortipelvis and dystonic posture that could regress during sleep. Some children appeared ataxic when attempting to walk during torticollis. 

In Table 1, all the essential clinical data from 15 authors are summarized.

A total of 273 cases reported by 15 authors were evaluated. In 59% of the described cases, the torticollis lasted from a few hours to a few days; by contrast, in 41% of cases, it persisted for more than 1 week. In only 4.5% of cases was a familial history of BPT detected. Family history was positive for migraine in a range between 25% and 100% of the cases described by Greene [5], Giffin [17], and Rosman [27] and it was positive for kinetosis in 54% and for migraine and/or kinetosis in 83% of cases. Usually, onset was in the first few months of life, 5–8 months, and initial episodes were usually longer and more frequent, regressing by the age of 3–5 years. Perhaps this is due to immaturity of the central nervous system and neurotransmitters in the first few months and years of life. BPT is often accompanied by symptoms like some features of migraine: vomiting, ataxia, pallor, irritability, apathy, and drowsiness. Subsequently, the syndrome resolves spontaneously and may be replaced by more migrainous features [5,6,7,12,14,17,26,27].

Greene [5] found that 63 out of 73 parents (90%) suffered from migraines and 2 from hemiplegic migraines (HMs). Brodsky [13] found the evolution of BPT to benign paroxysmal vertigo of childhood (BPVC) in 42.9% of cases and the evolution of BPVC to vestibular migraine (VM) in 15.4% of cases. Two of fourteen patients progressed through all three disorders. Prolonged torticollis episodes and abnormal rotary chair testing were associated with a higher risk of progression from BPT to BPVC. Greene [5] showed that 19% of the patients observed (14/73 pt) developed migraines (median age 9.25 years, range 2.5–23) and 63% (*n* = 46) developed another episodic syndrome associated with migraine. The patients who developed migraines were greater in number among those with any migraine symptoms during BPT attacks versus those without. The reported symptoms were phonophobia (58 vs. 21%, *p* = 0.02), photophobia and phonophobia (55 vs. 23%, *p* = 0.05), and photophobia, phonophobia, and motion sensitivity (60 vs. 22%, *p* = 0.02). On the basis of symptom duration, Drigo [6] classified two types of BPT. In the first type, the episodes lasted several hours or days (“periodic torticollis”) and, in the second type, the episodes lasted only a few minutes and were accompanied by ocular signs (“paroxysmal”). Hadjipanayis [7] and Greene [5] classified BPT on the basis of the duration of episodes as “Typical” 6 days (1 min–28 days), “Shortest” 2 days (1 min–10 days), and “Longest” 9 days (2 min–42 days).

- Etiology and Genetics

The etiopathogenesis of BPT is unknown. Many different underlying disorders involving vestibular and cerebellar structures, immaturity of the brain, or even channelopathy were proposed [27]. Surface electromyography (EMG) recordings showed continuous electrical discharge from the sternocleidomastoid in BPT, confirming that torticollis is a dystonia [28].

BPT is considered one of the “episodic syndromes that may be associated with migraine” in the International Classification of Headache Disorders, Third Edition (ICHD-3) [9]. The current understanding of the clinical phenotype of BPT is based largely on case reports and case series. BPT patients often have a family history of migraine. Mutations and polymorphisms in genes including Calcium Channel Voltage-dependent P/Q Type Alpha 1A subunit (CACNA1A) [14], Proline-rich transmembrane protein 2 gene (PRRT2) [17], and ATPase Na+/K+ Transporting Subunit Alpha 2 (ATP1A2) [14] have been identified in families with BPT. These genes encode ion channels or transmembrane proteins involved in cell signaling. Interestingly, the same and new mutations of these genes are also known to cause familial hemiplegic migraine (FHM) and other paroxysmal disorders, such as epilepsy, episodic ataxia, paroxysmal tonic upgaze, alternating hemiplegia (AH), and paroxysmal dyskinesia. The frequency of mutations in CACNA1A and PRRT2 in BPT is not known [17]. In 2002, Giffin et al. were the first to document a mutation in CACNA1A in two of four cases of atypical BPT [17]; a father and son had the same CACNA1A mutation linked to FHM, with associated ataxia. These mutations in the P/K-type calcium neural channel subunit (CACNA1A) were found in half of families with FHM, including those with cerebellar characteristics [17]. The author therefore hypothesized that the dystonia of the sternocleidomastoid muscle could be a form of atypical migraine aura. The four cases observed subsequently developed paroxysmal vertigo (BPVC) and episodes of ataxia. The hypothesis of channelopathy [17] suggests that the cerebellar cortex where the CACNA1A gene is abundantly expressed probably contributes to the expression of BPT. In 2008, Cuenca-León [18] reported base changes in the CACNA1A gene in a cohort of Spanish cases with different migraine variants, including hemiplegic migraine (HM), basilar migraine (BM), and children periodic syndrome (CPS). He screened 27 Spanish patients with hemiplegic migraine (HM), basilar-type migraine, or childhood periodic syndrome (CPS) for mutations in these genes. Two novel CACNA1A variants, p.Val581Met and p.Tyr1245Cys, and a previously annotated change, p.Cys1534Ser, were identified in individuals with HM, although they have not yet been proven to be pathogenic. Interestingly, p.Tyr1245Cys was detected in two patients displaying a changing, age-specific phenotype that began as BPT evolving into benign paroxysmal vertigo of childhood and later HM. None of these patients suffered from paroxysmal diseases at the time of the first and subsequent follow-up assessments. In the study group examined by the author, with a median follow-up of 13 years, 5 out of 11 children developed migraines, abdominal migraines (AMs), or cyclic vomiting (BCV). Among patients who experienced onset in childhood with episodes of BPV (*n* = 4), one developed HM, one BM, and two continued to display BPVC at the ages of 5 and 6 years, the latter with accompanying headaches. All BPVC patients had normal electroencephalogram (EEG) and audiometric testing; clinical screening of vestibular function in school-aged children was also normal. In 2009, Rosman et al. [27]. reported a neuromotor assessment of 10 children with BPT, and 5 showed gross motor delay during the period, with recurrent attacks of torticollis, while the motor delay remained persistent in four cases with parents migraineurs. In 2013, Marta Vila-Pueyo [19] described a genetic variant of two siblings with BPT that led to loss of function in CACNA1A. The mutation of CACNA1A, p.Glu533Lys, in the two brothers was also present in the presumably asymptomatic mother. This was the fourth mutation described in BPT related to the α subunit of the CaV2.1 neuronal channel encoded by the CACNA1A gene. This mutation induces a loss of channel function due to an altered gating with subsequent reduction of the ion passage of the P/Q channels. In 2016, Shin [20] examined the gene CACNA1A in a cohort of eight patients with BPT and their families. She found four different polymorphisms of the CACNA1A gene as well as, in one patient, the same polymorphism in the father and, in another patient, the same polymorphism in the mother. These polymorphisms are present in more than 0.2% of the reference population of the Center for Human Genetics of Cambridge. She found no pathogenic mutation and found no mutation or familiarity with hemiplegic migraine, episodic ataxia, or tonic paroxysmal upward gaze. She then hypothesized that CACNA1A mutations were more likely in children with BPT and a positive family history of migraine. In 2018, Danielson [14] found in a patient a variant with an unclear clinical significance of the ATP1A2 gene (NM_000702), C. MPEG 73G>C (p.Gly758 Ala). This variant has been described in the past in FHM 1. In another woman, the author described a pathogenic variant of the gene CACNA1A (NM_023035) C. 5176G>A (p. Val1726 Met). In 2018, Humbertclaude [21] reported gene mutations in CACNA1A (encoding the a-subunit of the neuronal P/Q-type calcium channel) in some cases of BPT, associated with other paroxysmal disorders such as paroxysmal deviation of the gaze (BTU) and BPV, suggesting a common pathophysiological basis of these three episodic syndromes. She studied mutations of the CACNA1A gene and the KCN1A gene in 50 families that had paroxysmic disorders such as BPT, BTU, and BPV. The presence of two more different episodic neurological syndromes in relatives was significantly associated with a CACNA1 mutation (*p* = 0.04). CACNA1A gene mutations in 4 out of 26 patients with BPT were as follows: CACNA1A (c.6783+3G>C; NA); CACNA1A (c.146dupA; 50AfsX26); CACNA1A (c.146dupA; p.Q50AfsX26); CACNA1A (c.2206C>T; p.Q736X). 

Table 2 summarizes the modifications of the CACNA1A gene described by the authors who studied benign paroxysmal torticollis.

- Diagnosis

The diagnosis of BPT is mainly based on the clinic. A correct medical history and physical examination are essential for diagnosis. A careful clinical story and a detailed description, as well as video recordings [27] and home videos, are essential. Diagnosis is an exclusion diagnosis and is confirmed when a pattern of stereotyped episodes is recognized. 

In Table 3, the ICHD-III diagnostic criteria are indicated.

A differential diagnosis must be made with respect to organic and neurological pathologies such as gastroesophageal reflux, idiopathic torsional dystonia, complex partial epilepsy, and all malformities conditions and space-occupying processes in the posterior cranial fossa, as well as cervical vertebral anomalies and craniovertebral junction anomalies. Neurological examination, electroencephalogram (EEG), and brain imaging tests are generally normal in patients with BPT and may help in differential diagnoses [7,27]. In some cases, at the first presentation, it may be necessary to exclude these secondary and quoad vitam dangerous forms. At the time of diagnosis, the family must be reassured that BPT is a benign pathology that tends to disappear spontaneously.

- Therapy

There is both an attack therapy and a preventive one, even if the majority of children do not need to take drugs [6]. Attack therapy is based on anti-inflammatory or pain-relieving drugs aimed at improving the child’s discomfort. Greene [5] in his large study reported that 64% of his 73 patients had used drugs for the acute treatment of BPT episodes. The most used acute medications were ibuprofen (41%), acetaminophen (41%), ondansetron (12%), and diphenhydramine (10%). These drugs had poor efficacy and patients’ responses were specified as follows: acetominophen ineffective in 17 out of 30 subjects, effective in 2 out of 30 subjects; ibuprofen ineffective in 13 out of 30 subjects, effective in 7 out of 30 subjects; ondansentron effective in 5 subjects out of 30 subjects, ineffective in 2 out of 30 subjects; and diphenhydramine ineffective in 5 out of 30 subjects, effective in 1 out of 30 subjects. The author identified a “Possibly useful” category, which indicated an uncertain benefit or an inability to remember a benefit, and this category represented 50% of the subjects treated. Only a minority of patients used other medications, which included caffeine (*n* = 3), cyproheptadine (*n* = 2), prochlorperazine (*n* = 2), metoclopramide (*n* = 1), lorazepam (*n* = 1), clonidine (*n* = 1), and cannabidiol injections (*n* = 1). A total of 16% of patients used preventive drugs and 50% reported benefits. The remaining 50% did not complete the therapeutic cycle. Another author reported four patients who showed a positive response to topiramate treatment [29].

## 4. Discussion

According to the criteria and classification of the ICHD-III, 2018 [10], BPT represents, together with infantile colic, a very early manifestation of the migraine condition. However, its pathogenesis is not clear and univocal. Based on the literature, a clinical spectrum of BPT can be outlined, which includes, at one extreme, the simpler and more benign forms that have no specific evolution, and, at the other, the forms that are accompanied by altered motor development. These forms are often linked to genetic mutations of the CACNA1A gene and PRRT2 and can evolve into hemiplegic migraine and basilar migraine, with consequent alteration of the quality of life. This is observed in the longitudinal and genetics studies of Danielson, Greene, and Humbertclaude, which highlight the characteristics of neurodevelopment in the patients observed [5,14,21]. The most frequent evolution of BPT is benign paroxysmal vertigo in childhood, an evolution found by several authors [6,12,17]. This is intriguing for the pathogenetic interpretation of BPT. BPT and BPVC are vestibular disorders considered to be of migraine origin, although they present clinically very differently and, typically, they occur in distinct age groups. It is unclear whether these disorders are caused by the same pathophysiological process with different symptoms at different ages or whether these are entirely distinct entities. Brodsky [13] described a number of patients showing progression from BPT to BPVC and from BPVC to VM. This phenomenon has been called “vestibular march”, similar to the phenomenon “atopic march”, a term used in pediatric allergology. A peripheral origin of BPT is also suggested by the abnormal response to the rotating chair test found by some authors such as in VM and BPVC [2,6]. It is possible that the type of BPT progressing towards BPVC may have a more peripheral vestibular pathophysiological model than cases of isolated BPT or isolated BPVC, which may be migraine forms from the beginning. Snyder [2] suggested in the first observation of BPT that peripheral vestibular dysfunction may be the cause, as in paroxysmal vertigo in childhood. This was supported by the fact that attacks in 4 of his 12 patients evolved into benign paroxysmal vertigo (BPVC) when children had grown up. The sternocleidomastoid dystonia demonstrated by Kimura [28] can be considered an atypical and prolonged motor aura (when it persists for more than 1 h). This can occur as an initial symptom accompanied or not accompanied by other symptoms of migraine, such as vomiting and pallor, in the same individual and in the same way as migraine aura may occur. Mutations in the α12.1 subunit of the CACNA1A gene have been found in BPT and in more than half of the families with FHM, including those with cerebellar characteristics [17]. The cerebellar cortex, where the gene is abundantly expressed, likely contributes to the expression of dystonic episodes. However, BPT in childhood is a benign and self-limiting disorder, although the neurodevelopment of affected patients is not always normal. It constitutes an important cause of paroxysmal movement disorder or dyskinesia in childhood. Child neurologists are well trained to distinguish between these benign events and serious neurologic causes. BPT is the first manifestation of a migraine condition along with infant colic. The concept that migraine is an age-dependent disorder is therefore reiterated. On the other hand, the phenotypic spectrum of mutations in the CACNA1A gene has been associated with three neurological phenotypes: familial and sporadic hemiplegic migraine type 1, episodic ataxia type 2, and spinocerebellar ataxia type 6.21. The vestibular instability, probably due to an altered calcium ion channel expressed in the inner ear and brain, could lead to reversible depolarization of hair cells, resulting in otoneurologic symptoms found in both migraine and periodic syndromes [30]. The coexistence of typical migraine syndromes does not occur rarely with the succession of clinical manifestations according to the age of the child [12]. In this regard, it should be remembered that structural changes in the brains of children and adolescents with migraines have been described. Abnormalities of the nociceptive pathway and cortical thickness have been described in children with migraines older than 12 years when compared to younger patients [31]. 

## 5. Conclusions

In summary, the following conclusions can be drawn:

1. There is a broad spectrum of clinical manifestations of benign paroxysmal torticollis (BPT), which must be correlated to the broad spectrum of migraines at later ages.
2. The diagnosis of BPT is underestimated. It is important that pediatricians know how to recognize the pathology to frame it correctly and direct parents to a correct diagnostic process.
3. Child neurologists are familiar with these conditions and know that paroxysmal non-epileptic events are extremely common in childhood; they come to their attention regularly, with an estimated incidence of 6.69 per 10,000 live births [32].
4. Longitudinal clinical studies supported by genetic, neuroradiological, and neuro-physiological studies can clarify the characteristics of the various forms of BPT and how peripheral or central it is in its pathogenesis, as is done today for adult migraines.

We have described the clinical manifestations of BPT and its evolution in over 273 cases reported in the literature, a number higher than in previous reviews on this topic.

Prospective longitudinal studies are useful to further characterize the nature of pediatric migraine variants and the relationships between them. The challenge will be to frame this context at a very early age with the aim of making the diagnosis of a migraine state in the first months of life.

## Figures and Tables

**Table 1:** Clinical data from 15 selected authors.

	Snyder [2] 1969	Deonna and Martin [18] 1981	Hanukoglu [19] 1984	Bratt and Menelaus [20] 1992	Drigo [6] 2000	Giffin [14] 2002	Al-Twaijri [12] 2002	Fernandez- Esqueban [1] 2006	Rosman [15] 2009	Hadjipanayis [7] 2015	Zlatanovic [21] 2017	Brodski [17] 2017	Danielsson [16] 2018	Moavero [11] 2019	Greene [5] 2021
**Number of cases**	12	5	4	4	22	4	11	18	10	3	12	14	12	33	73
**Male**	4	2	1	1	12	-			6	1	6	5	4	-	-
**Female**	8	3	3	3	10	-			4	2	6	8	8	-	-
**Age of onset**	2–30 m	2–12 m	7 d–10 m	2–7 m	1–9 m	3–6 m	2–36 m	2–12 m	5–4.5 m	<1–3 m	5–8 m	8–11 m	1–19 m	5 m	2–48 m
**Age of episode cessation**	10–60 m	30–36 m	36–48 m	6–120 m	<36 m	18–48 m			10–32 m	36–48 m	60 m		4 m–48 m		5–156 m
**Duration**	10 min–4 d	1 h–15 d	5 h–5 d	6 h–7 d	Min–14 d	15 min–1 h			5 d–10.5 d	A few days to 2 weeks	2 h 5 w	65+/−58 h	2–21 d	4 h–28 d	2–24 d
**Vomiting during episodes**	7/12	2/5	3/4	2/4	-	4/4	-	14	2/10	2/3	3	-	7	26	56
**Pallor**	-	1/5	-	1/4	-	1/4		12	1/10	2/3	4		3	26	47
**Ataxia**	4/12	5/12	3/4	3/4	-	1/4		14	2/10	1/3	1		4		58
**Motor delay**	-	-	-	-	no	-			8/10	2/3		11		-	-
**Behavioral changes**	7/12	4/5	3/4	2/4	-	4/4		3	4/10	3/3	9		4	26	39
**Family history of migraine**	-	4/5	1/4	-	13/22	4/4	8	6	10/10	3/3	10		5	29	63 2 (FME)
**Evolution to migraine**					6		2				2		3	17	27

d: days; h: hours; m: months; min: minutes; y: years; -: no data available.

**Table 2:** Changes in the CACNA1A gene in patients affected by benign paroxysmal torticollis described in the literature.

Authors	Patient Number	Mutation/Polymorphisms CACNA1A	Same Mutation/Polymorphism in Family Member
Giffin, 2002 [17]	2 (father and son)	Mutation p.1854X	Father
Cuenca-Leon, 2008 [18]	3	Mutation p.Tyr1245Cys	No family members tested
Vila-Pueyo, 2014 [19]	2 (brothers)	Mutation p.Glu533Lys (p.E533K)	Mother asymptomatic
Shin, 2016 [20]	4	Polymorphism exon 19 p.E992V, exon 18 p.E731A, exon 19 p.E1014K	Father, father, mother, mother
Danielson, 2018 [14]	1	Mutation p.Val1726Met	No family member testing
Humbertclaude, 2018 [21]	4	Mutation c.6783+3G>C; NA c.146dupA;p.Q50AfsX26 c.146dupA;p.Q50AfsX26; c.2206C>T; p.Q736X.	Mother

**Table 3:** International classification of headache disorders (ICHD-III): diagnostic criteria of benign paroxysmal torticollis.

Diagnostic Criteria of Benign Paroxysmal Torticollis
A. Recurrent attacks in a young child who satisfies criteria B and C
B. Episodes of head tilt to one side (both sides), with or without slight rotation, which resolve spontaneously within minutes or hours
C. At least one of the following symptoms or signs in association with the disorder: (1) paleness; (2) irritability; (3) feeling of malaise; (4) vomiting; (5) ataxia
D. Normal neurological examination in the interictal period
E. Not attributed to another disorder

## Data Availability

The authors confirm that the research data used for this review will be made available.

## Abbreviations

Benign paroxysmal torticollis (BPT); cyclic vomiting syndrome (CVS); abdominal migraine (AM); benign paroxysmal vertigo of childhood (BPVC); vestibular migraine (VM); International Classification of Headache Disorders, Third Edition (ICHD-3); basilar migraine (BM); children periodic syndrome (CPS); hemiplegic migraine (HM); electromyography (EMG); electroencephalogram (EEG); familial hemiplegic migraine (FHM); Calcium Channel Voltage-dependent P/Q Type Alpha 1A subunit (CACNA1A); Proline-rich transmembrane protein 2 gene (PRRT2); ATPase Na+/K+ Trans-porting Subunit Alpha 2 (ATP1A2); Sodium Voltage-gated channel Alpha subunit 1 (SCN1A), Preferred Reporting Items for Systematic reviews and Meta-Analyses (PRISMA).
